# circWWC3 enhances the progression of triple-negative breast cancer by interacting with vimentin to regulate the secretion of CSF2

**DOI:** 10.3389/fimmu.2025.1665608

**Published:** 2025-10-08

**Authors:** Ming Wu, Yifan Du, Yuyang Dong, Yang Zheng, Lina Gu, Xiaojun Tang, Li Yan, Hong Ji, Yang Sang, Fei Liu

**Affiliations:** ^1^ Department of Histology and Embryology, Hebei Medical University, Shijiazhuang, Hebei, China; ^2^ Research Center, the Fourth Hospital of Hebei Medical University, Shijiazhuang, Hebei, China; ^3^ The Department of Chemistry, College of Sciences, Shanghai University, Shanghai, China; ^4^ Department of General Surgery, The Second Hospital of Hebei Medical University, Shijiazhuang, Hebei, China; ^5^ Laboratory Animal Center, the Fourth Hospital of Hebei Medical University, Shijiazhuang, Hebei, China

**Keywords:** triple negative breast cancer, circWWC3, vimentin, epithelial- mesenchymal transition, CSF2

## Abstract

**Introduction:**

Circular RNAs (circRNAs) have been reported to be important in the development and progression of breast cancer. Nevertheless, the biological functions and mechanisms underlying the action of circRNAs in triple-negative breast cancer (TNBC) remain poorly understood. The present study aimed to explore the role of hsa_circ_0001910 (also termed circWWC3) interacting with vimentin in regulating the secretion of Colony Stimulating Factor 2 (CSF2) and its effects on the malignant biological behavior of triple-negative breast cancer as well as the cytotoxic activity of NK cells.

**Methods:**

RNA-Seq was utilized to investigate potential circRNAs involved in five pairs of breast cancer (BC) tissues and their corresponding normal tissues. Fluorescence *in situ* hybridization (FISH) was conducted to verify the relationship between circWWC3 expression and patient clinical pathological parameters, as well as its intracellular localization. Gain- and loss-of-function assays were conducted to investigate the biological functions of circWWC3 in TNBC. A microarray analysis of mRNA expression profiles was conducted to explore the downstream target genes of circWWC3. RNA pull-down assays, RNA immunoprecipitation (RIP), and mass spectrometry were carried out to uncover the proteins interacting with circWWC3. Rescue experiments were performed to investigate the potential regulatory role of circWWC3 in the progression of TNBC *in vivo* and in virto.

**Results:**

In our present study, Circular RNA sequencing analysis revealed that the expression of circWWC3 was significantly upregulated in breast cancer (BC). FISH assay results indicated that circWWC3 is highly expressed in TNBC, and its elevated expression is associated with the patient’s T stage and lymph node metastasis, and it is primarily localized in the cytoplasm. The results of gain- and loss-of-function assays indicate that knockdown of circWWC3 significantly suppressed the proliferation, invasion, and migration of TNBC cells, while enhancing the killing efficiency of NK-92MI cells against TNBC cells. In contrast, overexpression of circWWC3 exhibited the opposite effects. The microarray analysis data indicated that CSF2 may be a downstream target of circWWC3. Interaction of circWWC3 with vimentin and their downstream target genes was confirmed by RNA pull-down, RIP, and mass spectrometry. Rescue experiments confirmed that vimentin knockdown partially counteracted the tumor-promoting effects of circWWC3. Further analysis revealed that circWWC3 upregulates CSF2 secretion mainly through its interaction with vimentin, a core component of the Epithelial-mesenchymal transition (EMT) signaling pathway, thereby facilitating the malignant progression of TNBC.

**Conclusion:**

Overall, our findings reveal that elevated expression of circWWC3 serves a role in the malignant progression of TNBC by directly interacting with the S56 phosphorylation site of vimentin, an interaction that is associated with increased secretion of CSF2. Furthermore, circWWC3 emerges as a potential biomarker for breast cancer diagnosis and presents an attractive therapeutic target for the treatment of TNBC.

## 1 Background

Breast cancer is among the most common malignancies affecting women, serving as the leading cause of cancer-related mortality amongst this demographic (1). Triple-negative breast cancer (TNBC) is characterized by the absence of estrogen receptors (ER), progesterone receptors (PR) and the lack of human epidermal growth factor receptor 2 (HER2) overexpression/amplification, which accounts for 15-20% of all breast cancer cases (2). Previous clinical data have confirmed that TNBC exhibits significant tumor heterogeneity and does not benefit from anti-estrogen treatments or anti-HER2 targeted therapies. Therefore, identifying effective therapeutic targets for TNBC is an urgent issue that warrant a clinical solution.

Circular RNAs (CircRNAs) represent a category of RNA molecules that are generated through the head-to-tail splicing of precursor mRNAs synthesized from host genes (3). Their circular structure confers stability, rendering them less susceptible to degradation by RNAases (4). Additionally, circRNAs are prevalent in eukaryotic organisms, with certain circRNAs exhibiting higher abundance compared with their linear counterparts (5). These aforementioned characteristics of circRNAs provide opportunities and insights for cancer treatment and detection (6). A number of studies have previously found that circRNAs can serve a role in the development of malignant tumors. In bladder cancer cells, abnormal nuclear export of circ-nuclear receptor co-repressor 1 was found to inhibit lymphangiogenesis and lymph node metastasis by regulating the TGFβ/Smad signaling pathway (7). In intrahepatic cholangiocarcinoma, the circular RNA circ-zinc finger protein 215 was found to promote tumor growth and metastasis through inactivation of the PTEN/AKT signaling pathway (8). In esophageal cancer, circ-trinucleotide repeat containing adaptor 6B can suppress the proliferation and invasion of esophageal cancer cells by regulating microRNA (miR or miRNA) 452-5p/dystroglycan (9). In addition, another previous study has found that the circular RNA circ-cytochrome P450 family 24 subfamily A member 1 can bind to pyruvate kinase 2 (PKM2) to regulate the NF-κB signaling pathway, promoting chemokine (CC motif) ligand 5 secretion to facilitate the progression of esophageal squamous cell carcinoma (10). The circular RNA circWWC3 was reported to promote M2 macrophage polarization and breast tumor immune escape by regulating IL-4 expression and secretion, thereby enhancing cancer progression (11). Furthermore, there is accumulating evidence that circRNAs can contribute to the onset and development of TNBC (12, 13).

Epithelial-mesenchymal transition (EMT) is a reversible cellular process that mainly takes place during embryonic development, tissue repair and the advancement of cancer (14). During EMT, epithelial cells transform into a spindle-shaped, mesenchymal phenotype and gain enhanced mobility (15). This process is crucial for the progression and metastasis of various types of cancer (16, 17). Accumulating evidence indicates that circRNA-mediated EMT can mediate a role in the initiation and progression of malignancies. Twist, an EMT transcription factor, has been reported to enhance the expression of circ-Cullin2 (Cul2) by interacting with the promoter of Cul2, leading to the binding of this circular RNA to specific miRNAs and subsequently increasing vimentin expression in hepatocellular carcinoma (18). In bladder urothelial carcinoma, circ-protein arginine N-methyltransferase 5 (PRMT5), derived from the PRMT5 gene located on chromosome 14q11.2, was demonstrated to facilitate EMT and enhance tumor aggressiveness through the circPRMT5/miR-30c/SNAIL1/E-cadherin axis (19). In papillary thyroid cancer, circ-nucleoporin 214 can act as an oncogenic molecule by sponging miR-145 to upregulate zinc finger E-box binding homeobox 2 expression, thereby promoting cell proliferation, migration and invasion (20).

In the present study, circWWC3 (hsa_circ_0001910) was identified in BC tissues through RNA sequencing. The expression of circWWC3 was strongly associated with the clinicopathological characteristics of TNBC patients and was highly upregulated in both TNBC cells and tissues. Research performed *in vitro* and *in vivo* indicated that circWWC3 is key in facilitating the proliferation and metastasis of TNBC cells. Mechanistically, circWWC3 promoted the proliferation, colony formation, migration and invasion of TNBC cells, which was partially mediated by the induction of colony stimulating factor 2 (CSF2) secretion. Furthermore, it has been established that circWWC3 can promote EMT and facilitate the progression of TNBC by interacting with vimentin. Consequently, the present study demonstrated that increased expression levels of circWWC3 can trigger the EMT pathway through its interaction with vimentin, leading to the enhanced secretion of CSF2 and accelerated malignant progression of TNBC. Taken together, these findings suggest a novel function for circWWC3 to serve as a potential biomarker for prognosis assessment and a potential therapeutic target in TNBC.

## 2 Materials and methods

### 2.1 RNA-sequence and mRNA microarray analysis

The expression profiles of circRNAs in BC samples (n=5) and their corresponding adjacent non-tumorous tissues (n=5) were compared using RNA-Seq. These adjacent tissue samples were from the same patients as the tumors (paired samples), with tissues selected more than 2 cm away from the tumor margin serving as adjacent non-tumor tissue, and all samples were pathologically validated to ensure they were free of tumor cell infiltration. All RNA-Seq data comparisons were performed using a paired analysis method. RNA quality assessment and transcriptome analysis were performed by Guangzhou Geneseed Biotech Co., Ltd. Total RNA was extracted using TRIzol reagent (Invitrogen; Thermo Fisher Scientific, Inc.), with quality verified by NanoDrop 2000 (A260/280>1.8, A260/230>2.0) and Agilent 2100 Bioanalyzer (RNA Nano 6000 Kit; RIN>7.0). Libraries were prepared using Total RNA-seq (H/M/R) Library Prep Kit for Illumina^®^ (Vazyme), quantified to 3.2 nM using Qubit 4.0 Fluorometer (dsDNA HS Assay Kit) and KAPA Library Quant Kit (Roche), then sequenced in 150 bp paired-end mode on Illumina NovaSeq 6000 platform. For bioinformatics analysis, the reads were mapped to the genome using STAR (https://github.com/alexdobin/STAR) and DCC (https://github.com/dieterich-lab/DCC) was used to identify circRNAs and estimate circRNA expression. TMM (trimmed mean of M-values) was used to normalize gene expression. Differentially expressed genes were identified using the edgeR program (v3.36.0) with a false discovery rate (FDR) threshold of <0.05 and |log2FC|>1. No covariates (such as age, sex, or disease stage) were included in the statistical model. For the analysis of cell mRNA microarrays, total RNA was extracted in triplicate from both the negative control MDA-MB-231 cells and the MDA-MB-231 cells with circWWC3 knockdown. The assessment of RNA quality and transcriptome analysis were conducted by Shanghai OE Biotech Co., Ltd. Differentially expressed genes were identified using thresholds of |log2FC|≥ 1 and *p* < 0.05.

### 2.2 Specimens and tissue microarray

In total, five paired fresh frozen tumor samples and their adjacent normal tissues were collected from patients with BC at the Breast Center of the Fourth Hospital of Hebei Medical University, with informed consent obtained from the patients. These patients had not received any treatment prior to surgery. The tissue microarray containing 150 TNBC tissues and 30 normal adjacent tissues (cat. no. HBreD180Bc01) were obtained from the Shanghai Xinchao Biological Technology Co. Ltd. The present study received approval from the Ethics Committee of the Fourth Hospital of Hebei Medical University (approval no. 2023KY045). All methods were performed in accordance with the relevant guidelines and regulations.

### 2.3 Cell culture and transfection

Normal human mammary epithelial cells MCF-10A and four types of human TNBC cells, namely MDA-MB-231, BT-549, MDA-MB-468 and HS-578T, were obtained from Procell Life Science & Technology Co., Ltd. MCF-10A cells were cultured in a specialized epithelial culture medium (Procell, China). MDA-MB-231 and HS-578T cells are cultured in DMEM, whilst MDA-MB-468 cells were cultured in Leibovitz’s L-15 medium (GIBCO; Thermo Fisher Scientific, Inc.) and BT-549 cells were grown in RPMI 1640 medium (GIBCO; Thermo Fisher Scientific, Inc.). All four triple-negative breast cancer cell lines are cultured with 10% FBS and supplemented with 1% penicillin and streptomycin. Additionally, HS-578T and BT-549 cells are supplemented with 10 μg/ml insulin. All cell lines were maintained at 37˚C in a humidified incubator with 5% CO2. For transfection, circWWC3 small interfering (si)RNA (sense: 5’-GAAAGAGGATAACAAAGCC-3’; antisense: 5’- GGCUUUAUUACUCUUUC-3’), the si-NC (sense: 5’-CGUACGCGGAAUACUUCGA-3’; antisense: 5’-UCGAAGUAUUCCGCGUACG-3’), and vimentin overexpression (pCMV-VIM(human)-EGFP-Neo) constructs were obtained from Haixing Biosciences. Overexpression constructs for circWWC3 and the control (pLC5-ciR) vector were sourced from Guangzhou Geneseed Biotech Co., Ltd. A total of 3 μg plasmid DNA was transfected using FuGENE HD (Promega Corporation) for the overexpression experiments. For siRNA transfection, MDA-MB-231 and BT-549 cells were transfected with HiPerFect (Qiagen GmbH) according to the manufacturer’s protocols.

### 2.4 RNase R treatment and actinomycin D assay

Total RNA was extracted from MDA-MB-231 and BT-549 cells using TRIzol reagent (Invitrogen; Thermo Fisher Scientific, Inc.). In the actinomycin D assay, MDA-MB-231 and BT-549 cells were seeded in 6-well plates prior to treatment with actinomycin D (AdooQ Bioscience, USA). All cells were exposed to 2 μg/mL actinomycin D for durations of 4, 8, 12, and 24 hours, while the control group remained untreated. For the RNase R assay, 2 μg RNA was treated with 3 U/μg RNase R (Epicentre; Illumina, Inc.) at 37°C for 15 min, followed by a 3-min incubation at 85°C. A parallel control sample was processed identically but in the absence of RNase R. Following the RNase R and actinomycin D treatments, the expression levels of circWWC3 and linear WWC3 were assessed using reverse transcription-quantitative reverse transcription PCR (RT-qPCR).

### 2.5 Quantitative reverse transcription PCR validation

Total RNA was extracted from the cells using TRIzol reagent (Invitrogen; Thermo Fisher Scientific, Inc.). cDNA was synthesized using GoScript™ (Promega Corporation). qRT-PCR was conducted using SYBR Green Reagent (Promega Corporation). The primer sequences used for PCR amplification are provided in **Table 1**. U6 snRNA and GAPDH served as internal controls for circRNAs and mRNAs, respectively.

**Table 1:** The primers used in the PCR amplification.

Genes	Primers
circWWC3	Forward, 5’-CTTCAAGAACAGCTGCTCCG-3’ Reverse, 5’-CCAACACAATCGGCAAAGGT-3’
WWC3	Forward, 5’-TAGCAAGTCGTCGGGATAGG-3’ Reverse, 5’-CGCCTCATCCAGTTTGTAGC-3’
CSF2	Forward, 5’-TCCTGAACCTGAGTAGAGACAC-3’ Reverse, 5’-TGCTGCTTGTAGTGGCTGG-3’
KLRC3	Forward, 5’-GCCAGCATTTTACCTTCCTCA-3’ Reverse, 5’-TCTGATGCACTGCAAGCTCAA-3’
SH2D1B	Forward, 5’-CGAATCTTCAGAGAGAAACACG-3’ Reverse, 5’-GGGCTGGTTCTCTTTATTGG-3’
TNFRSF10D	Forward, 5’-GTTGGCTTTTCATGTCGGAAGA-3’ Reverse, 5’-CCCAGGAACTCGTGAAGGAC-3’
ULBP3	Forward, 5’-TCTATGGGTCACCTAGAAGAGC-3’ Reverse, 5’-TCCACTGGGTGTGAAATCCTC-3’
KLRC1	Forward, 5’-AGCTCCATTTTAGCAACTGAACA-3’ Reverse, 5’-CAACTATCGTTACCACAGAGGC-3’
PTPN6	Forward, 5’-GGTGTCCACGGTAGCTTCC-3’ Reverse, 5’-ACAGGTCATAGAAATCCCCTGAG-3’
GAPDH	Forward, 5’-CGCTGAGTACGTCGTGGAGTC-3’ Reverse, 5’-GCTGATGATCTTGAGGCTGTTGTC-3’
U6	RT, 5’-AACGCTTCACGAATTTGCGT-3’ Foward, 5’-CTCGCTTCGGCAGCACA-3’ Reverse, 5’-AACGCTTCACGAATTTGCGT-3’

### 2.6 Nuclear-cytoplasmic fraction

Cytoplasmic and nuclear RNA isolation was performed using the Thermo Scientific NE-PER nuclear and cytoplasmic extraction kit (Thermo Fisher Scientific, Inc.) according to the manufacturer’s protocol. Briefly, cells were treated with cell fractionation buffer and incubated on ice for 5 min before centrifugation. This process facilitated the separation of the cytoplasmic fraction from the nuclear pellet. Subsequently, the samples were incubated at 25°C with a lysis/binding solution to differentiate the nuclear and cytoplasmic RNA. The RNA for both fractions was then isolated using a filter cartridge and eluted with an elution solution. The isolated samples were analyzed using RT-qPCR.

### 2.7 Fluorescence *in situ* hybridization

The Cy3-labeled circWWC3 probe was purchased from Geneseed Biotech Co., Ltd. (Guangzhou, China), and the FISH assay was performed using a commercial kit (Geneseed Biotech) following the manufacturer’s protocol. For cell-based FISH, MDA-MB-231 and BT-549 cells were grown on coverslips, fixed in 4% formaldehyde (20 min, RT), and permeabilized with 0.2% Triton X-100 in PBS. For tissue FISH, TNBC tissue microarray slides were treated to remove paraffin using xylene and ethanol solutions, followed by a 10-min incubation with proteinase K at 37°C for blocking. The FISH probes were diluted to 20 nM, denatured, and pre-hybridized before application to cells or tissues. Hybridization was carried out overnight at 37°C. After hybridization, nuclei were counterstained with DAPI, and images were captured using a confocal laser scanning microscope (Carl Zeiss AG). The sequences of the probes are as follows: circWWC3-Cy3 5’Cy3-TCAATGGCTTTGTTATCCTCTTTCT-3’Cy3.

### 2.8 Cell proliferation and colony formation assay

Cell proliferation was assessed using the Cell Counting Kit-8 (CCK-8) assay. MDA-MB-231, BT-549, MDA-MB-468 and HS-578T (2x10³ cells per well) cells were seeded into a 96-well plate, before 10 μl CCK-8 solution was added to each well daily. After 1 h incubation at 37°C, the absorbance at 450 nm was measured using a multifunctional microplate reader. For colony formation assays, MDA-MB-231, BT-549, MDA-MB-468 and HS-578T cells were stored in 6-well plates at the density of 2,000 cells per well. The cells were then placed in a 37°C sterile incubator for 12 days. Afterwards, the cell colonies were stained with crystal violet solution and subsequently observed.

### 2.9 Transwell assays

Cell migration and invasion abilities were assessed using a Transwell chamber (Corning, Inc.). For invasion assays, Matrigel (BD Biosciences) was evenly spread in the chamber at 37°C overnight. Migration assays were performed using Transwell chambers without Matrigel. Cells (4x10^4^) were placed into the upper chambers containing serum-free culture medium, whilst the lower chamber was filled with culture medium supplemented with 10% FBS. After 24–48 h, the cells in the upper chamber were wiped off and the MDA-MB-231, BT-549, MDA-MB-468 and HS-578T cells that had invaded to the bottom of the membrane were stained with crystal violet. Cells were counted in five randomly selected fields per membrane using ImageJ software (v1.53; National Institutes of Health). The mean value from three independent experiments was used for statistical analysis.

### 2.10 Enzyme linked immunosorbent assay

The protein expression levels of CSF2 in cell supernatants were quantified using a CSF2 Human ELISA Kit (ProteinTech Group, Inc.) following the manufacturer’s protocols.

### 2.11 RNA pull−down and mass spectrometry assays

The RNA pull-down probe targeting the junction site of circWWC3 was synthesized by Guangzhou RiboBio Co., Ltd. MDA-MB-231 and BT-549 cells were first lysed using a lysis buffer and subsequently incubated with the biotin-labeled circWWC3 probe (circWWC3 probe/5’BiosG/TCAATGGCTTTGTTATCCTCTTTCT/3’Bio/). The cell lysates were then combined with streptavidin-coated agarose magnetic beads to capture the biotin-labeled RNA-protein complexs, utilizing the Pierce™ Magnetic RNA-Protein Pull-Down Kit (Thermo Fisher Scientific, Inc.). After incubation, the beads were collected using a magnetic stand, washed three times with Wash Buffer to remove nonspecifically bound proteins, and the RNA-binding proteins were eluted by incubation with Elution Buffer (containing competitive ligands or denaturing agents) at room temperature for 15 min with gentle agitation. The proteins isolated by probes were identified via Western blot analysis. Further mass spectrometry analysis was performed using QLBio (Beijing, China). Proteins were identified and quantified using the Proteome Discoverer Software 2.4 (Thermo Fisher Scientific). The mass spectrometry data were searched against the UniProtKB Human database using the Sequest HT search engine. The search parameters were set as follows: trypsin as the enzyme with up to two missed cleavages allowed; precursor mass tolerance of 15 ppm and fragment mass tolerance of 0.02 Da; carbamidomethylation (C) was set as a fixed modification; oxidation (M) and acetylation (protein N-terminus) were set as variable modifications. To ensure high-confidence identifications, the false discovery rate (FDR) was controlled at ≤ 1% at both the peptide-spectrum match (PSM) and protein levels using the Percolator node. Protein identifications were required to contain at least one unique peptide.

### 2.12 RNA FISH-immunofluorescence

MDA-MB-231 and BT-549 cells were seeded onto glass cover slips and fixed at room temperature using 4% paraformaldehyde, followed by a 1-h rehybridization step. The cells were then treated overnight at 37°C with a Cy3-labeled circWWC3 probe. After blocking with 10% BSA (cat. no. A8010; Solarbio, Beijing, China) at 37°C for 30 mins, the cells were incubated for 1 h at room temperature with a vimentin antibody (cat. no. 10366-1-AP; ProteinTech Group, Inc.), followed by a 1-h incubation at 37°C with an Alexa Fluor™ 488-conjugated secondary antibody (cat. no. SA00013-2; ProteinTech Group, Inc.). Finally, the coverslips were sealed with parafilm-containing DAPI (cat. no. SI103-11; Seven, Beijing, China) and images were captured using a confocal microscope.

### 2.13 Western blot analysis

MDA-MB-231, BT-549, MDA-MB-468 and HS-578T cells from each group were lysed using RIPA lysis buffer (cat. no. SW104-2; Seven, Beijing, China) supplemented with PMSF (1 mM). The resulting protein lysates were denatured through heat treatment and subjected to electrophoresis on 10% SDS polyacrylamide gels. After separation, the proteins were transferred onto PVDF membranes and blocked with 5% skimmed milk. Primary antibodies against E-cadherin (cat. no. 20874-1-AP; 1:1,000; ProteinTech Group, Inc.), N-cadherin (cat. no. 22018-1-AP; 1:1,000; ProteinTech Group, Inc.), Vimentin (cat. no. 10366-1-AP; 1:1,000; ProteinTech Group, Inc.), phosphorylated (p-)-Vimentin (S56; cat. no. ab217673; 1:1,000; Abcam) and β-actin (cat. no. 20536-1-AP; 1:1,000; ProteinTech Group, Inc.) were added and incubated overnight at 4°C. Following this, secondary antibodies (cat. no. SA00001-1/SA00001-2; 1:5,000; ProteinTech Group, Inc.) were applied for 1 h. The membranes were developed using an ECL kit (Beijing Solarbio Science & Technology Co., Ltd.). The intensity of the immunoblot lanes was quantified using the Image J software (v1.53; National Institutes of Health).

### 2.14 RNA Immunoprecipitation assay

RIP assays were conducted using a commercial RIP Kit (Geneseed, Guangzhou, China) according to the manufacturer’s protocol. In brief, MDA-MB-231 cells transfected with Flag-Vimentin-WT or Flag-Vimentin-S56A plasmids for 48 hours were collected (2×10^7^ cells), lysed, and centrifuged. The resulting supernatants were incubated overnight at 4°C with magnetic beads conjugated with either anti-Flag antibody or control IgG (5 μg per sample; Proteintech, China). Subsequently, RNA was isolated from the immunoprecipitated complexes, and relative expression levels of target RNAs were measured via RT-qPCR.

### 2.15 Lactate dehydrogenase assay

The cytotoxic activity of NK-92MI cells toward TNBC cells was evaluated by measuring LDH release with the CytoTox 96^®^ Non-Radioactive Cytotoxicity Assay Kit (cat. no. G1780, Promega, Madison, USA). Briefly, breast cancer cells were transfected with the following constructs: si-NC, si-circWWC3, si-NC + vector, si-circWWC3 + vector, and si-circWWC3 + vimentin. After 24 hours, NK-92MI cells were co-cultured with the transfected TNBC cells at effector-to-target ratios of 5:1, 10:1, and 20:1 for 6 hours. Subsequently, the culture supernatants were collected and analyzed for LDH activity.

### 2.16 *In vivo* tumorigenesis assays

The *in vivo* experiments received approval from the Institutional Animal Care and Use Committee of the Fourth Affiliated Hospital of Hebei Medical University (approval no. IACUC-4th Hos Hebmu-2023045). All BALB/c nude mice were sourced from Hfk Bioscience Co., Ltd. (Beijing, China). In the xenograft tumor study, 4-week-old female BALB/c nude mice were randomly assigned (n=5 per group) to one of three experimental groups: i) MDA-MB-231 cells stably transfected with shControl + Vector; ii) shcircWWC3 + Vector; or iii) shcircWWC3 + Vimentin, with the experimenter blinded to the treatment regimen during all measurements. The cells were then injected subcutaneously into the right mammary fat pad of each mouse (5x10^6^ cells in 100 μl PBS) to establish the xenograft model. To ensure ethical compliance, tumors were allowed to grow until they reached a maximum volume of 1.5 cm³. After tumor formation, the volume was measured every 3 days to calculate tumor growth. At the end of the experiment, the mice received an intraperitoneal injection of ketamine (100 mg/kg) in combination with xylazine (10 mg/kg), both prepared in physiological saline. The mice were then humanely euthanized by cervical dislocation, and tumors were harvested and weighed. All the experiments were conducted following the ARRIVE guidelines.

### 2.17 Molecular-docking analysis

The structural configurations of circWWC3 and vimentin protein were computationally predicted using RNACOMPOSER (v1.0; http://rnacomposer.ibch.poznan.pl/) and AlphaFold2 (v2.3.2; https://www.alphafold.ebi.ac.uk/), respectively, followed by structural optimization in AutoDock Vina (v1.1.2; http://vina.scripps.edu/) through protonation state adjustment, atomic completeness restoration, and motif reconstruction. Molecular docking analysis was then performed using HDOCK (v1.1; http://hdock.phys.hust.edu.cn/) with the protein structure maintained as rigid body under comprehensive surface scanning conditions, generating 100 docking conformations from which the most thermodynamically favorable complex (determined by minimal binding energy score) was selected for three-dimensional visualization and analysis using PyMOL (v2.4.0; https://pymol.org/2/).

### 2.18 Immunohistochemical analysis

Immunohistochemistry (IHC) was employed to evaluate protein expression in tumor tissue specimens. Following deparaffinization of tumor sections, endogenous peroxidase activity was blocked with 3% hydrogen peroxide. The sections were then incubated overnight at 4°C with primary antibodies targeting CSF2 (cat. no. 17762-1-AP; 1:500; ProteinTech Group, Inc.), after which HRP-conjugated secondary antibodies were applied. Antigen visualization was achieved using diaminobenzidine (DAB), and hematoxylin was used for nuclear counterstaining. IHC assessment was conducted in a blinded manner, following the same scoring methodology applied in the FISH assay. Specimens were categorized as negative (0), weak (1–2), moderate (3), or strong (4–6) according to the composite scoring system.

### 2.19 Statistical analysis

Statistical analyses for all experiments were conducted using SPSS 24.0 (IBM Corp) and GraphPad Prism software (Dotmatics). Data are expressed as mean ± SD. Comparisons between groups were conducted using paired Student’s t-test or one-way analysis of variance (ANOVA) followed by Dunnett’s test or Tukey’s test. The χ2 test was used to assess the relationship between gene expression and the various clinicopathological characteristics. P<0.05 was considered to indicate a statistically significant difference.

## 3 Results

### 3.1 Characterization of circWWC3 in TNBC

**Figure 1:**
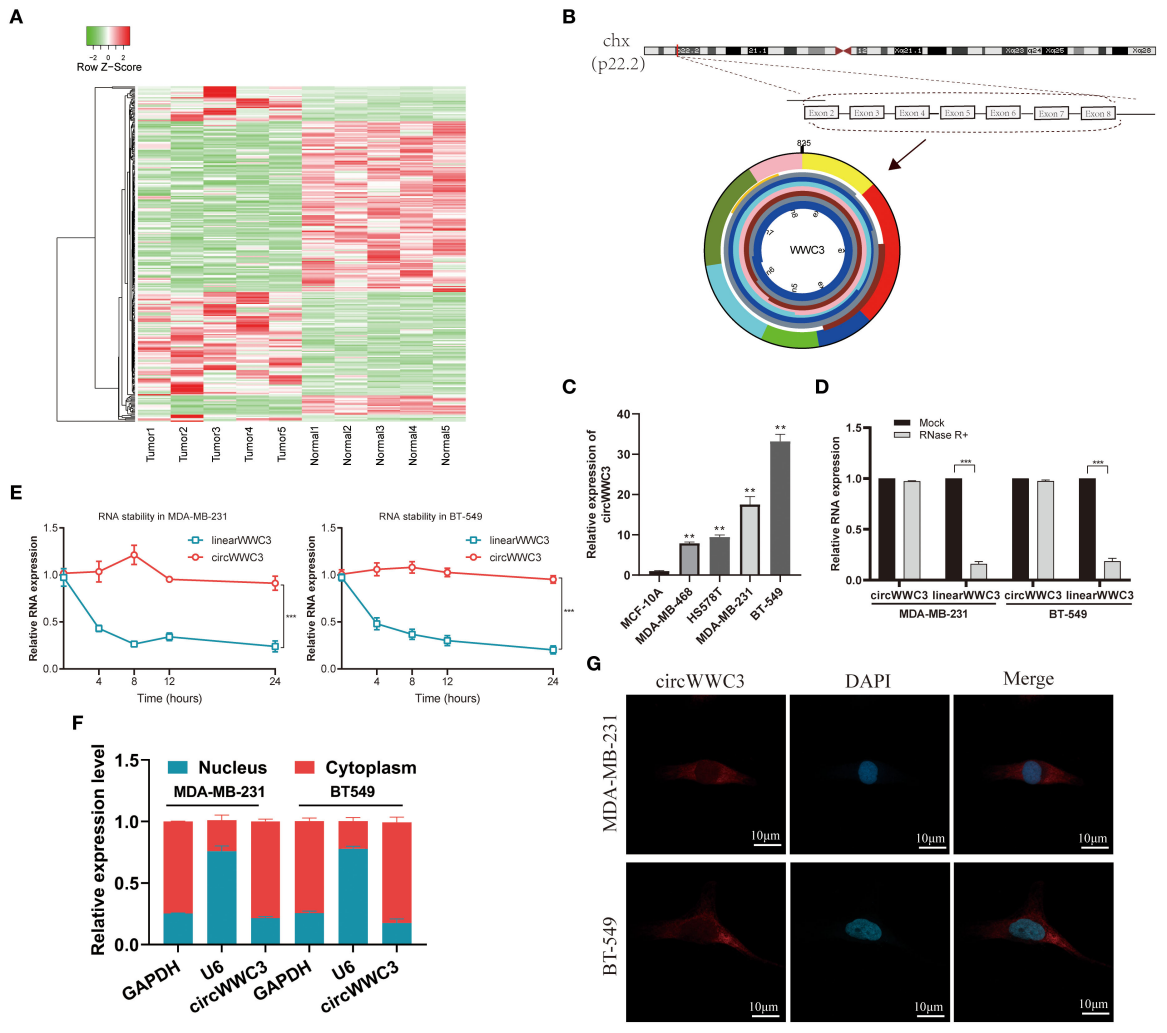
Expression profiles of circRNAs in TNBC and characterization of circWWC3. **(A)** Heatmap displaying the differentially expressed circRNAs in the five human BC tissues compared with adjacent non-tumor tissues. **(B)** circWWC3 is generated through the backsplicing of exons 2–8 of the WWC3 gene. **(C)** The expression levels of circWWC3 in breast cancer cell lines compared with normal breast epithelial cells. ^**^P<0.01. Relative RNA levels of circWWC3 and WWC3 mRNA in MDA-MB-231 and BT-549 cells following treatment with RNase R (D) and actinomycin (E) ***P<0.001. The nucleoplasmic separation experiment (F) and RNA FISH assay (G)demonstrating the subcellular localization of circWWC3 in MDA-MB-231 and BT-549 cells, with nucleoli stained using DAPI and circWWC3 labeled red with Cy3. Scale bars, 10mm. Experiments were repeated at three times (n = 3). Circ, circular RNA; TNBC, triple-negative breast cancer.

CircRNA expression patterns were examined in five paired samples of BC using the Illumina sequencing platform. Relative to normal tissues, the BC tissue group exhibited a notable upregulation of 224 circRNAs and a marked downregulation of 231 circRNAs (**Figure 1A**). Hsa_circ_0001910, derived from WWC3 and designated as circWWC3, was found to be significantly upregulated among the differentially expressed circRNAs. Therefore, circWWC3 was chosen as the primary focus of the present study.

circWWC3 originates from exons 2–8 of the host gene WWC3 (**Figure 1B**). Initially, the expression of circWWC3 was assessed in both normal breast epithelial cells (MCF-10A) and TNBC cells (MDA-MB-231, BT-549, MDA-MB-468 and HS-578T). MDA-MB-231 and BT-549 cells, which are TNBC cell lines, consistently demonstrated elevated expression of circWWC3 (**Figure 1C**). Based on the results of the actinomycin D and RNase R assays, circWWC3 exhibited greater stability compared with WWC3 mRNA following treatment with actinomycin D (**Figure 1D**) and RNase R (**Figure 1E**). Subsequent nuclear-cytoplasmic fractionation and FISH assays demonstrated that circWWC3 was predominantly localized in the cytoplasm instead of the nucleus of MDA-MB-231 and BT-549 cells (**Figures 1F, G**). These aforementioned findings indicated a significant increase in circWWC3 expression levels in both TNBC tissues and cells, where the primary localization was the cytoplasm in TNBC cells.

### 3.2 Expression of circWWC3 in TNBC tissues and its relationship with the clinical pathological indicators of patients

**Figure 2:**
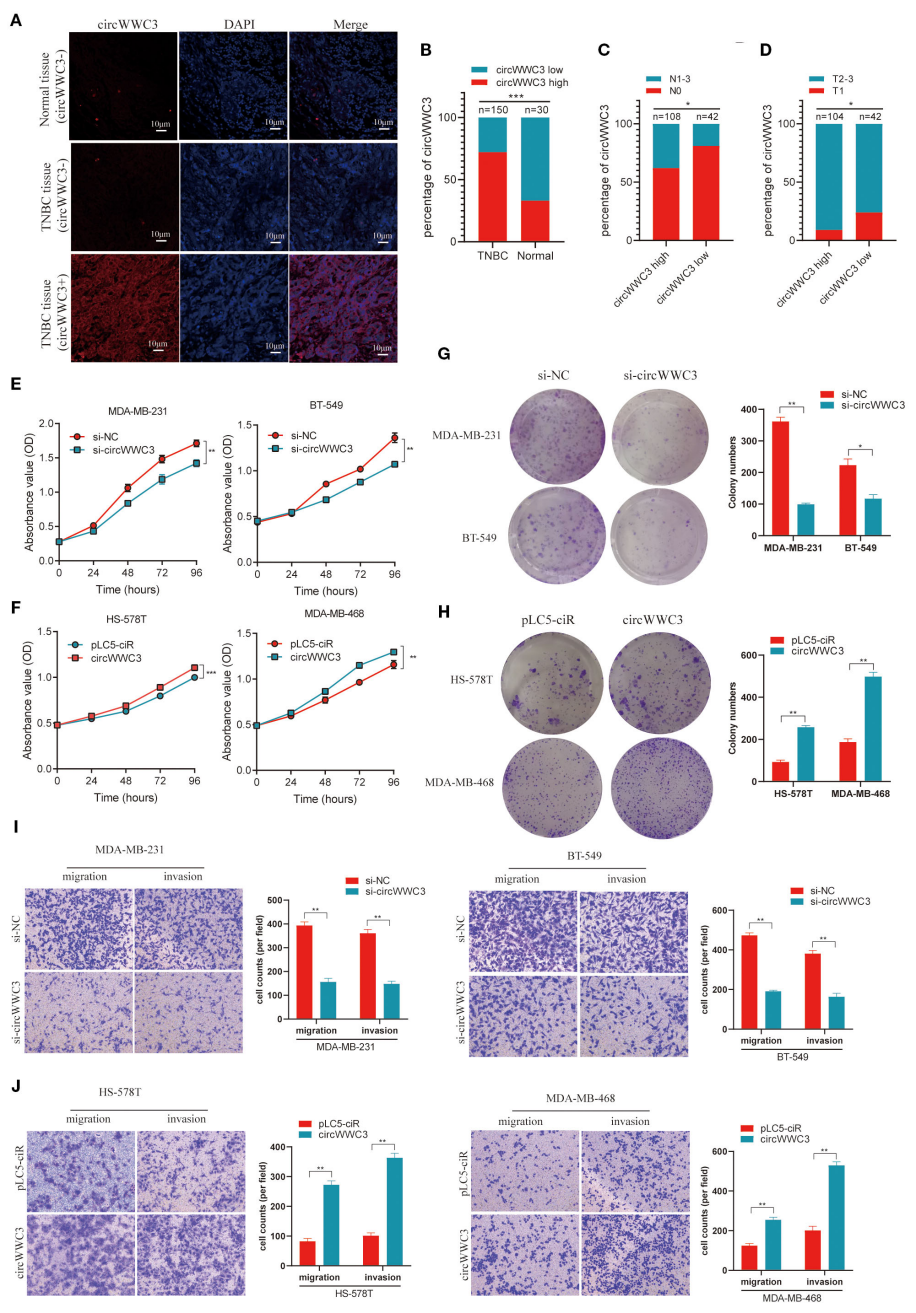
circWWC3 is associated with the progression of TNBC and promotes the proliferation, migration and invasion of TNBC cells. **(A)** RNA FISH analysis of tissue microarrays depicting the expression levels of circWWC3 in 150 TNBC tissues alongside 30 corresponding adjacent tissues. Scale bars, 10μm. **(B)** The percentage of circWWC3 expression in 150 TNBC tissues and 30 corresponding adjacent tissues. **(C)** The percentage of lymph node metastasis in 42 cases of TNBC tissues with low circWWC3 expression and 108 cases of TNBC tissues with high circWWC3 expression. **(D)** The percentage of T stage in 104 cases of TNBC tissues with high circWWC3 expression and 42 cases of TNBC tissues with low circWWC3 expression. ^*^P<0.05 and ^***^P<0.001. The proliferation ability of TNBC cells transfected with **(E)** si-circWWC3 or **(F)** circWWC3 overexpression plasmid was detected using the Cell Counting Kit-8 assay. ^**^P<0.01 and ^***^P<0.001. The clone formation ability of TNBC cells transfected with **(G)** si-circWWC3 or **(H)** circWWC3 overexpression plasmid was detected using a colony formation assay. ^*^P<0.05 and ^**^P<0.01. Transwell migration and matrigel invasion assays were used to evaluate the migration and invasion capabilities of TNBC cells transfected with either **(I)** si-circWWC3 or **(J)** a circWWC3 overexpression plasmid. ^**^P<0.01. Experiments were repeated at three times (n = 3). Circ, circular RNA; TNBC, triple-negative breast cancer; si, small interfering RNA.

To assess the expression level of circWWC3 in a larger population, a tissue microarray that included 150 TNBC tissues along with 30 corresponding adjacent tissues was obtained. It was found that the expression of circWWC3 was significantly increased in TNBC tissues compared with that in the corresponding adjacent tissues (**Figures 2A, B**). The association between circWWC3 expression levels and the clinicopathological characteristics of patients with TNBC was next examined. It was observed that patients with advanced T stage and lymph node metastasis demonstrated elevated levels of circWWC3 expression (**Figures 2C, D**) (**Table 2**). Analysis of the Kaplan-Meier plotter database also revealed that the expression of WWC3, the parental gene of circWWC3, is associated with poor patient prognosis (**Supplementary Figure S2**). These aforementioned results suggest that circWWC3 is highly expressed in TNBC, where its elevated expression is associated with the patient’s T stage and lymph node metastasis.

**Table 2:** Relationship between the expression level of circWWC3 and clinical features.

Group	N	circWWC3		χ^2^	*P*
		High	Low		
Histological grade				0.744	0.389
G1-2	39	26	13		
G3	111	82	29		
T stage				6.071	0.014
T1	19	9	10		
T2-3	127	95	32		
Lymph node status				4.919	0.027
N0	101	67	34		
N1-3	49	41	8		

### 3.3 circWWC3 enhances the proliferation, invasion and migration ability of TNBC cells

To further investigate the impact of circWWC3 on the biological functions of TNBC, gain-of-function and loss-of-function experiments were conducted. RNA interference was used to suppress circWWC3 expression and confirmed the knockdown efficiency in MDA-MB-231 and BT-549 cells (**Supplementary Figure S1A**). The CCK-8 and clone formation assay results indicate that circWWC3 knockdown significantly inhibited the proliferation and clone formation capabilities of MDA-MB-231 and BT-549 cells (**Figures 2E, G**). Transwell cell invasion and migration assays found that circWWC3 knockdown significantly inhibited the invasion and migration of MDA-MB-231 and BT-549 cells (**Figure 2I**).

The circWWC3 overexpression plasmid was then constructed, which were transfected into MDA-MB-468 and HS-578T cells. RT-qPCR analysis demonstrated that the expression level of circWWC3 was markedly increased upon circWWC3 overexpression (**Supplementary Figure S1B**). CCK8 assay and colony formation assay indicated that the overexpression of circWWC3 effectively enhanced the proliferation and clone formation capabilities of MDA-MB-468 and HS-578T cells (**Figures 2F, H**). The promoting effects of cell migration and invasion were also demonstrated using cell migration and invasion assays (**Figure 2J**). The aforementioned data collectively indicate that circWWC3 may contribute to the development and progression of TNBC.

### 3.4 CSF2 serves as a functional downstream mediator of circWWC3

**Figure 3:**
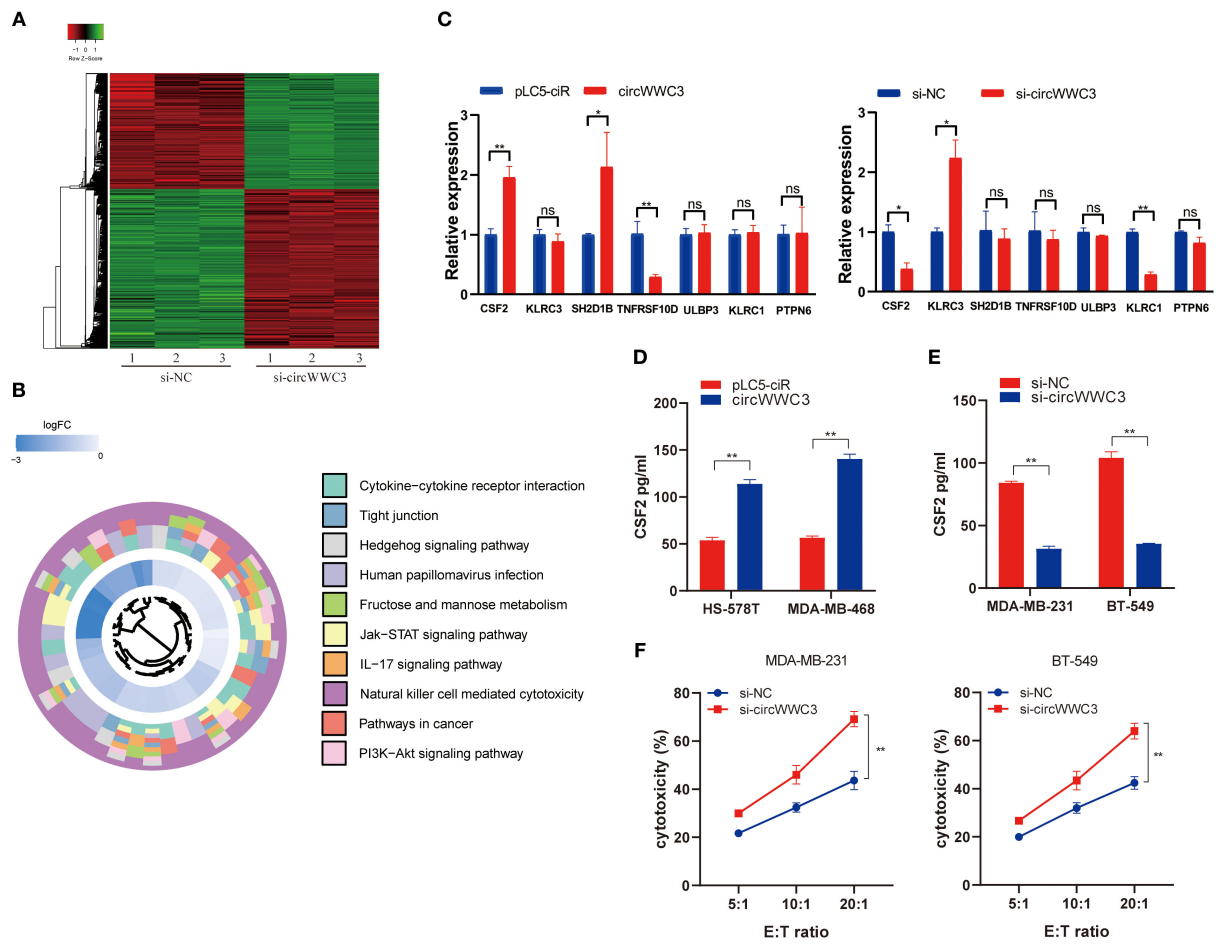
CSF2 acts as a functional downstream mediator of circWWC3.**(A)** The heatmap displays the differentially expressed mRNAs in MDA-MB-231 cells transfected with si-circWWC3. **(B)** Kyoto Encyclopedia of Genes and Genomes signaling pathway analysis of the target genes of circWWC3. **(C)** Relative expression levels of representative genes in BT-549 and HS-578T cells treated with si-circWWC3 or circWWC3 overexpression plasmid. ns P≥0.05, ^*^P<0.05 and ^**^P<0.01. **(D)** ELISA was used to determine the concentration of CSF2 in the supernatants of HS-578T cells and MDA-MB-468 cells with circWWC3 overexpression. ^**^P<0.01. **(E)** ELISA measured the levels of CSF2 in the supernatants of MDA-MB-231 cells and BT549 cells with circWWC3 knockdown. **(F)** The cytotoxicity of NK-92MI cells was assessed using the LDH assay after co-culture with circWWC3-knockdown MDA-MB-231 and BT-549 cells at effector-to-target ratios of 5:1, 10:1, and 20:1. ^**^P<0.01. Experiments were repeated at three times (n = 3). Circ, circular RNA; TNBC, triple-negative breast cancer; si, small interfering RNA; CSF, colony-stimulating factor.

To explore the downstream signaling pathways associated with circWWC3, an mRNA expression profile microarray analysis was next conducted in MDA-MB-231 cells transfected with si-negative control (NC) or si-circWWC3. These results indicate that after si-circWWC3 transfection, a total of 682 genes exhibited changes compared with the si-NC group, with 379 genes being upregulated and 303 genes downregulated (**Figure 3A**). The Kyoto Encyclopedia of Genes Genomes pathway enrichment analysis revealed that circWWC3 is involved in the regulation of cytokine-cytokine receptor interactions, tight junctions and natural killer cell-mediated cytotoxicity, among others (**Figure 3B**). A selection of representative genes was chosen and validated through RT-qPCR, with CSF2 being one of the genes that exhibited the most significant changes (**Figure 3C**). Several studies have previously identified CSF2 as a chemotactic factor for tumor cells, demonstrating its strong association with proliferation, invasion and metastasis (21, 22). Subsequent overexpression or knockdown experiments of circWWC3 indicated that overexpressing circWWC3 led to an increase in CSF2 levels (**Figure 3D**), whilst knocking down circWWC3 resulted in a decrease in CSF2 levels (**Figure 3E**), according to ELISA performed on the culture supernatants of TNBC cells. Notably, circWWC3 overexpression had minimal impact on CSF2 mRNA levels but significantly enhanced CSF2 protein secretion, suggesting that the regulation likely occurs at the post-translational or secretory level rather than at the transcriptional level.

**Figure 4:**
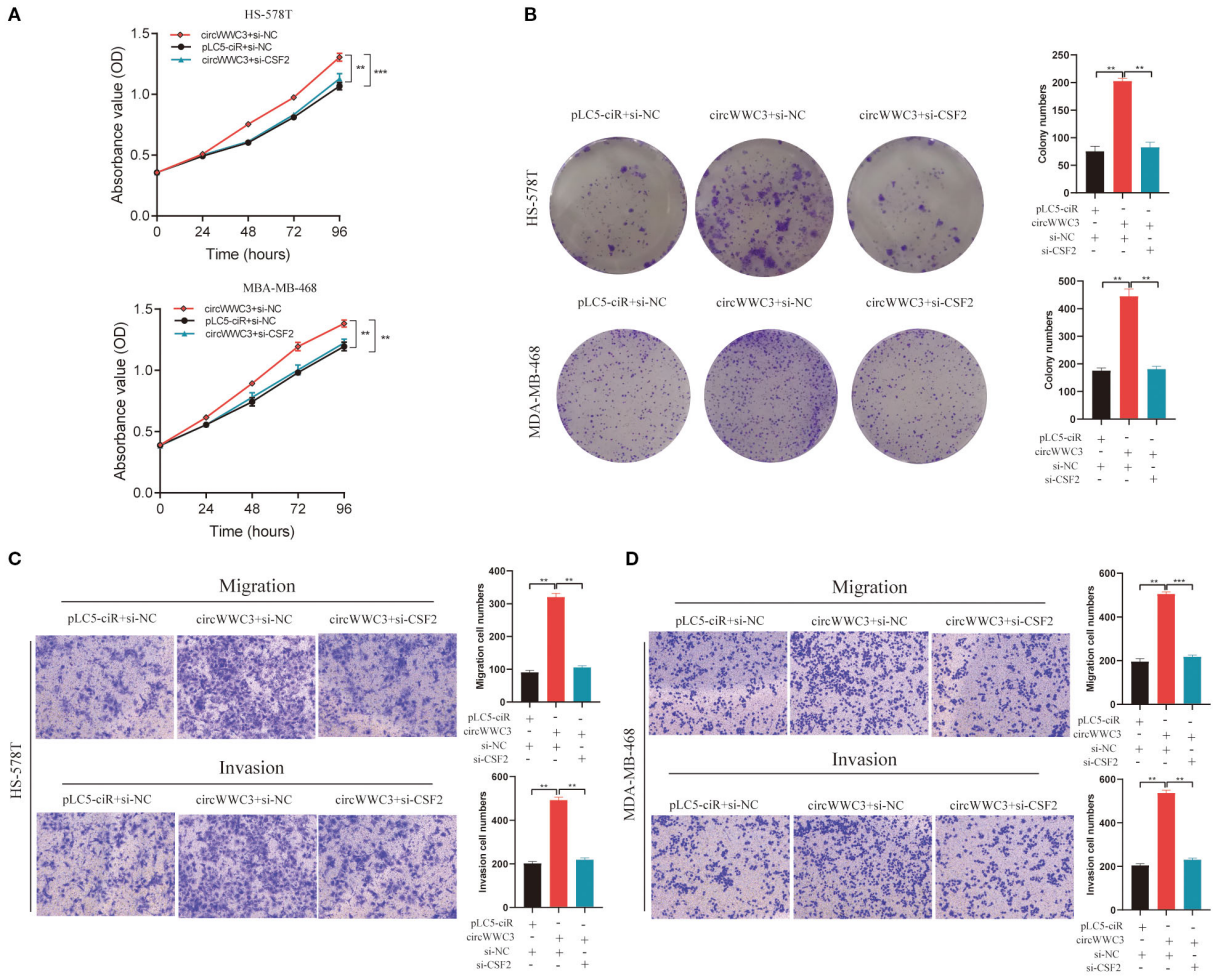
circWWC3 enhance the proliferation, colony formation, migration and invasion of triple-negative breast cancer cells by upregulating the expression of CSF2. Knockdown of CSF2 partly reversed the promotion of cell proliferation and colony formation induced by circWWC3 overexpression in **(A)** HS-578T and **(B)** MDA-MB-468 cells. ^**^P<0.01 and ^***^P<0.001. The silencing of CSF2 partially countered the enhanced cell invasion and migration stimulated by the overexpression of circWWC3 in both **(C)** HS-578T and **(D)** MDA-MB-468 cells. ^**^P<0.01 and ^***^P<0.001. Experiments were repeated at three times (n = 3). Circ, circular RNA; CSF, colony-stimulating factor.

As the KEGG pathway analysis indicated that circWWC3 is involved in NK cell-mediated cytotoxicity, we performed NK cell cytotoxicity assays. The results demonstrated that the cytotoxicity of NK-92MI cells was significantly enhanced when co-cultured with circWWC3-knockdown MDA-MB-231 and BT-549 cells compared to the si-NC group (**Figure 3F**). To verify whether the biological function of circWWC3 in TNBC cells is mediated by the stimulation of CSF2, rescue experiments involving circWWC3 and CSF2 were conducted. The results indicated that knockdown of CSF2 partially reversed the effects of circWWC3 overexpression on the proliferation (**Figure 4A**) and clonogenic ability (**Figure 4B**) of MDA-MB-468 and HS-578T cells. In addition, the results of the Transwell cell invasion and migration experiments indicated that knocking down CSF2 partially reversed the effects of circWWC3 overexpression on the invasion and migration abilities of MDA-MB-468 and HS-578T cells (**Figures 4C, D**). In summary, these results indicate that the overexpression of circWWC3 promotes the malignant progression of TNBC cells by enhancing the secretion of CSF2.

### 3.5 circWWC3 facilitates increased CSF2 secretion by interacting with vimentin

**Figure 5:**
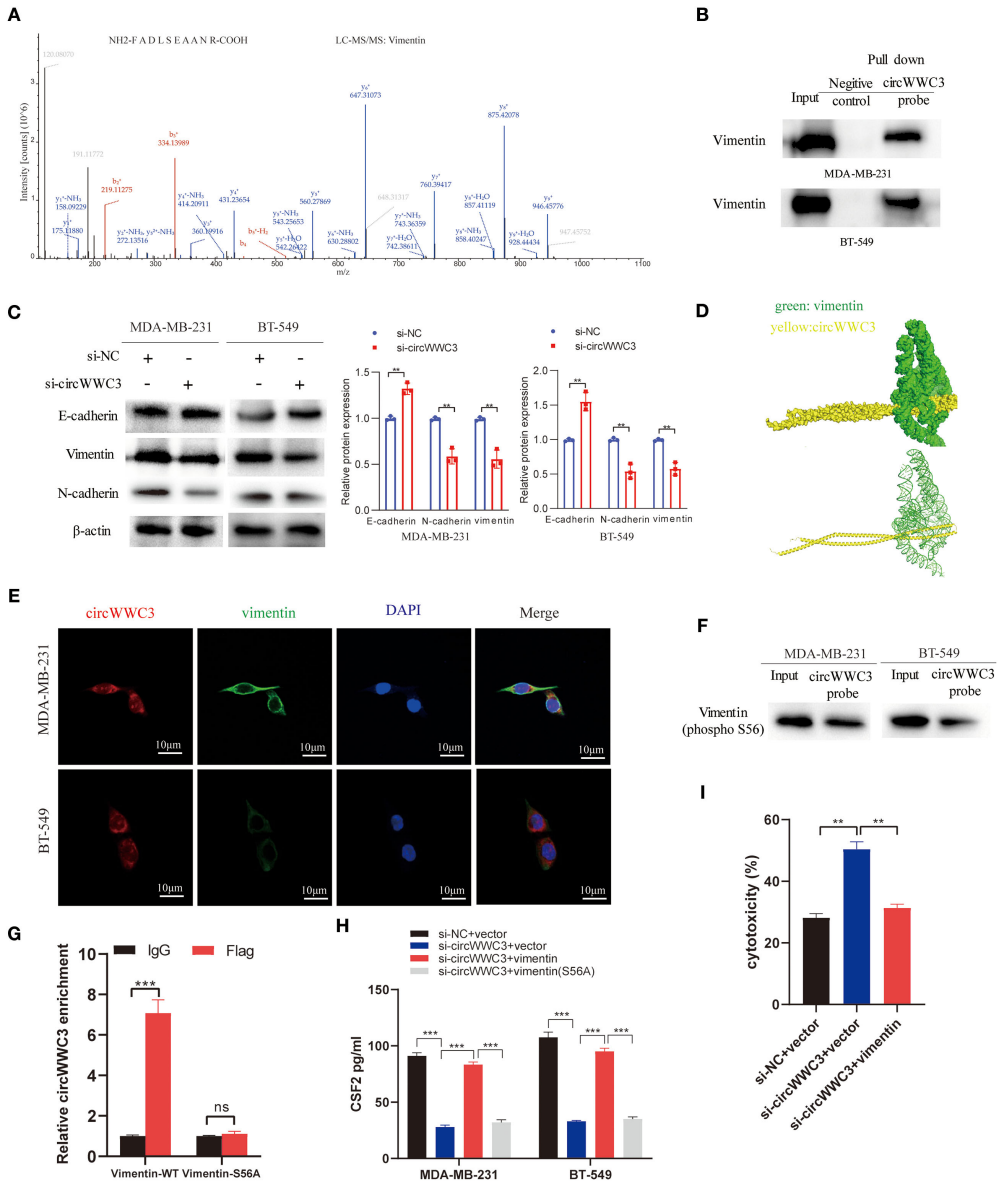
circWWC3 interacts with vimentin to regulate the secretion of CSF2. **(A)** Mass spectrometry assays revealed that the protein vimentin was pulled down by biotin-labeled circWWC3 from the lysates of MDA-MB-231 cells. **(B)** The binding of circWWC3 to vimentin was confirmed through RNA pull-down followed by western blot analysis. **(C)** The western blot analysis revealed alterations in the expression of EMT markers, including E-cadherin, N-cadherin and vimentin, following the knockdown of circWWC3. **(D)** Verification of the interaction between vimentin and circWWC3 was conducted through molecular docking analysis. The binding score of RNA to vimentin protein was -379.36 kcal/mol. The image illustrates the electrostatic surface characteristics of vimentin and circWWC3. **(E)** The RNA FISH-immunofluorescence technique revealed the co-localization of circWWC3 and vimentin in the MDA-MB-231 and BT-549 cell lines. **(F)** RNA pull-down and western blot assays demonstrating the binding sites of circWWC3 on vimentin. **(G)** RNA immunoprecipitation (RIP) assays confirm a Ser56-dependent interaction between circWWC3 and vimentin. **(H)** Secreted CSF2 protein levels in MDA-MB-231 and BT549 cells were measured by ELISA after circWWC3 knockdown, vimentin overexpression, or vimentin (S56A) mutant overexpression. **(I)** The cytotoxic activity of NK-92MI cells against MDA-MB-231 cells was assessed using an LDH release assay. ^**^P<0.01, ^***^P<0.001. Experiments were repeated at three times (n = 3). Circ, circular RNA; CSF, colony-stimulating factor.

**Figure 6:**
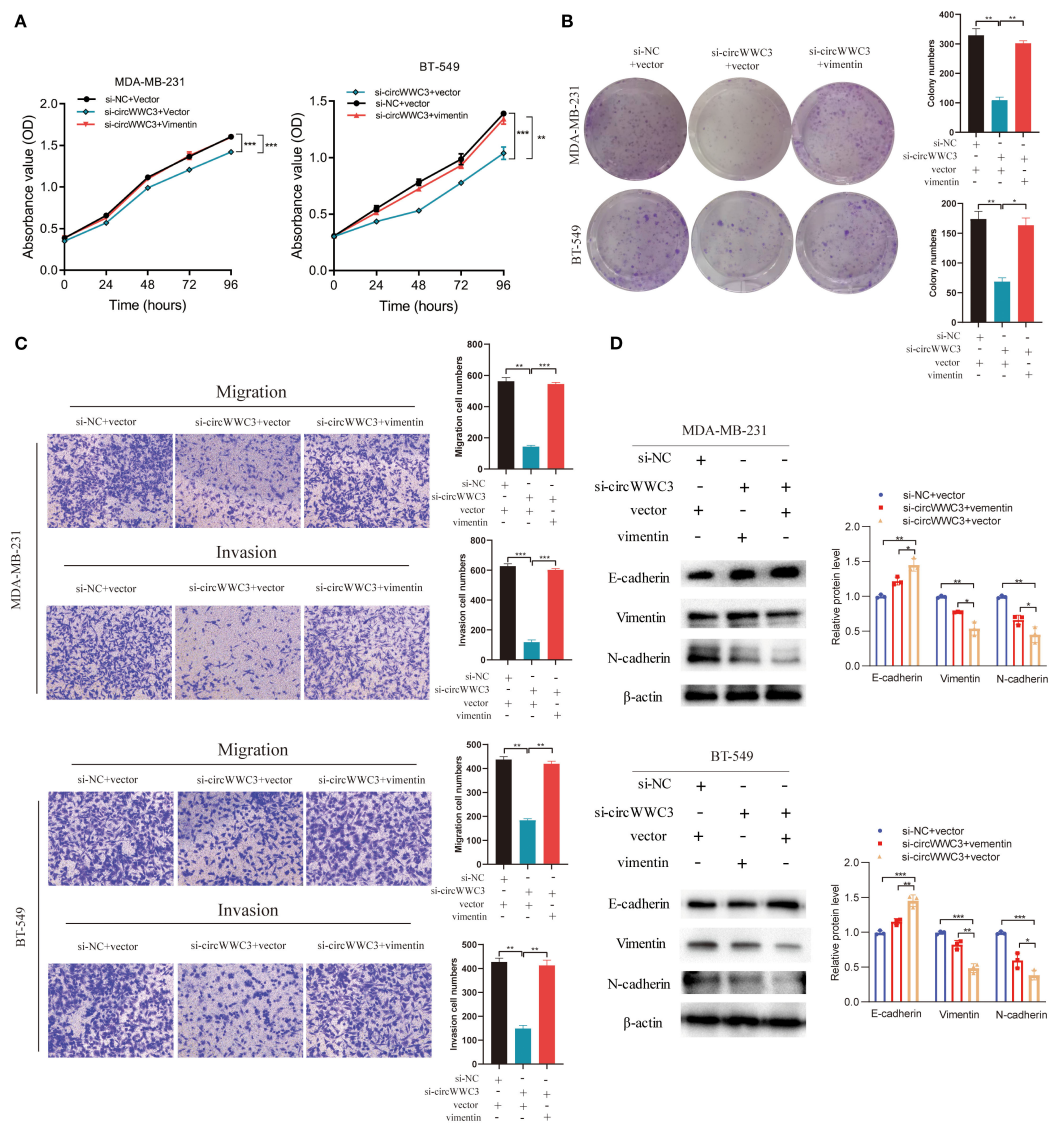
circWWC3 regulate vimentin expression to affect the proliferation, colony formation, migration, invasion and epithelial-mesenchymal transition of triple-negative breast cancer cells. **(A)** The proliferation ability of MDA-MB-231 cells and BT549 cells with circWWC3 knockdown or vimentin overexpression was detected by Cell Counting Kit-8 assays. ^**^P<0.01 and ^***^P<0.001. **(B)** The cloning ability of MDA-MB-231 cells and BT549 cells with circWWC3 knockdown or vimentin overexpression was assessed using colony formation analysis. ^*^P<0.05 and ^**^P<0.01. **(C)** The migration and invasion capabilities of MDA-MB-231 cells and BT549 cells with circWWC3 knockdown or vimentin overexpression were evaluated using Transwell migration and Matrigel invasion assays. ^**^P<0.01 and ^***^P<0.001. **(D)** The protein expression levels of E-cadherin, N-cadherin and vimentin were assessed by western blotting following circWWC3 knockdown or vimentin overexpression. Circ, circular RNA. Experiments were repeated at three times (n = 3).

circRNAs has been previously reported to serve a role in molecular regulation through their interactions with proteins (10). To investigate the proteins that can associate with circWWC3, RNA pull-down and mass spectrometry assay were conducted to identify its interacting proteins. An interaction between the vimentin protein and circWWC3 was observed (**Figure 5A**; **Table 3**), which was validated using western blot analysis (**Figure 5B**). Western blotting results indicated that knocking down circWWC3 markedly reduced the expression of vimentin and N-cadherin in MDA-MB-231 and BT-549 cells, whilst markedly increasing that of E-cadherin (**Figure 5C**). Molecular-docking analysis indicated that circWWC3 is folded to form a DNA-like double helix structure, which enhances the stability of the RNA. The folded circWWC3 encases the vimentin protein and aligns closely with it, facilitating the formation of a stable RNA-protein complex (**Figure 5D**). RNA FISH immunofluorescence analysis confirmed the co-localization of circWWC3 and vimentin in the cytoplasm of MDA-MB-231 and BT-549 cells (**Figure 5E**). Furthermore, the target sites for circWWC3 on vimentin were confirmed through RNA pulldown and western blotting using antibodies against p-vimentin (S56) (**Figure 5F**). RNA immunoprecipitation (RIP) assays validate that the binding of circWWC3 to vimentin is dependent on the Ser56 residue (**Figure 5G**). These results suggest that circWWC3 can bind to vimentin at the S56 site. A previous study has reported that circRNAs can bind to vimentin and influence the EMT in tumor cells upstream of tumor progression (23). Additionally, other studies have indicated that the EMT in breast cancer cells can enhance the secretion of CSF2 (24). Therefore, it was next hypothesized that this interaction may serve a role in the secretion of CSF2. Notably, the downregulation of CSF2 induced by circWWC3 knockdown was partially reversed upon vimentin overexpression. However, this reversal effect was abolished when vimentin was mutated to Vimentin (S56A) (**Figure 5H**). The NK-92MI cell cytotoxicity results demonstrated that the enhanced killing ability induced by circWWC3 knockdown could be counteracted by vimentin overexpression (**Figure 5I**). Furthermore, vimentin overexpression partially recovered the abilities of proliferation (**Figure 6A**) and colony formation (**Figure 6B**), which were originally inhibited by circWWC3 knockdown. Similarly, the suppressive effects on migration and invasion caused by the knockdown of circWWC3 were partially reversed through the overexpression of vimentin (**Figure 6C**). Moreover, the inhibition of EMT in TNBC induced by circWWC3 knockdown was found to be reversed by vimentin overexpression (**Figure 6D**).

**Table 3:** List of the 10 most abundant proteins coprecipitated with circWWC3 identified by MS.

Gene name	Abundances	MW [kDa]	Score
VIMENTIN	421345147.6	53.6	173.87
PKM	179497540.5	57.9	146.77
NCL	116308892.3	76.6	100.41
HRNR	42062609.31	282.2	84.3
ACACA	46519019.06	265.4	81.06
DSP	39442800.09	331.6	72.68
FLG2	22251742.38	247.9	67.17
LTF	39479051.85	78.1	65.12
AZGP1	57972853.06	34.2	61.6
HNRNPA2B1	212968419.7	37.4	56.1

**Figure 7:**
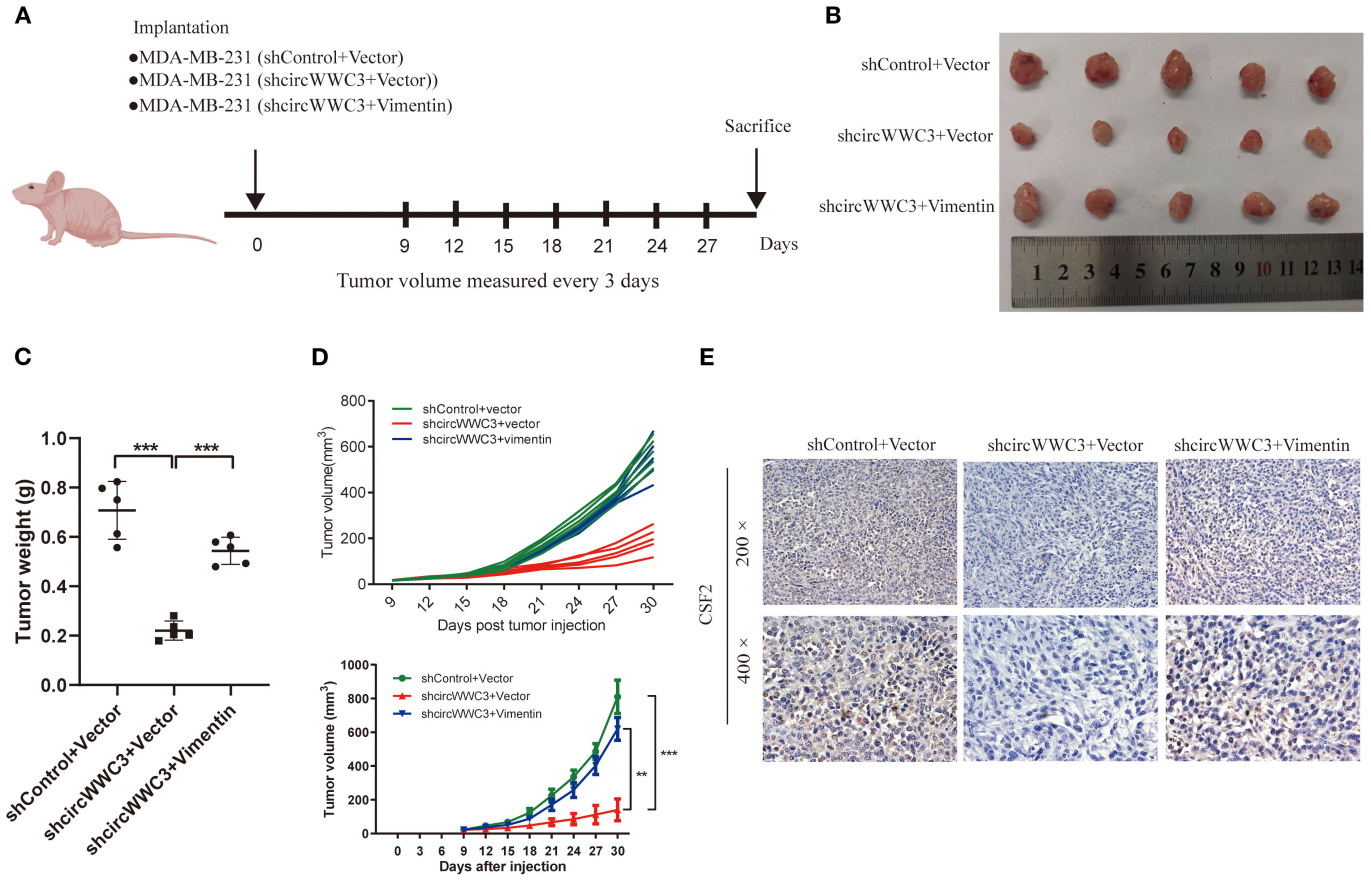
circular RNA WWC3 regulates the formation of xenograft tumors *in vivo* by modulating vimentin. **(A)** Schematic diagram of grouping for the xenotransplantation model. **(B)** Images of xenograft tumors obtained from different groups. **(C)** Tumor weight and **(D)** growth curves of subcutaneous xenograft tumors (n=5). ^**^P<0.01 and ^***^P<0.001. **(E)** The expression levels of CSF2 protein were detected in the different groups using immunohistochemistry. The data are presented as the mean ± SD (n=5).

To explore the relationship between circWWC3 and the growth of TNBC tumors, MDA-MB-231 cells stably transfected with shControl + vector, shcircWWC3 + vector or a combination of shcircWWC3 + vimentin vectors were subcutaneously injected into BALB/c nude mice. Knocking down circWWC3 significantly inhibited the growth of xenograft tumors. However, this inhibitory effect was reversed when vimentin was overexpressed (**Figures 7A–D**). We also examined the expression of CSF2 in the aforementioned groups using IHC. Our results indicated that CSF2 expression was markedly reduced upon circWWC3 knockdown, and this inhibitory effect was counteracted when vimentin was overexpressed simultaneously with circWWC3 knockdown (**Figure 7E**). Overall, these findings suggest that vimentin-mediated activation of the EMT signaling pathway may significantly influence the promoting effects of circWWC3 on the progression of TNBC, both *in vitro* and *in vivo*.

## 4 Discussion

TNBC is characterized by its notable aggressiveness, significant heterogeneity and poor prognosis (25, 26). Consequently, the identification of effective therapeutic targets for patients with TNBC is critical. circRNAs have garnered considerable attention due to their abundant expression, evolutionary stability, robust structure and diverse biological functions (27, 28). The malignant progression of tumors has been previously associated with the abnormal expression of circRNAs. circ-cofilin 1 has been documented to contribute to TNBC oncogenesis by enhancing the histone deacetylase 1/c-Myc/mutp53 signaling pathway, making it a potential diagnostic biomarker and therapeutic target for TNBC with TP53 mutations (13). Therefore, identifying novel functional circRNAs can enhance the understanding of mechanisms underlying the onset and progression of TNBC whilst revealing promising novel therapeutic targets.

In the present study, a potential circRNA (circWWC3) originating from exons 2–8 of the host gene WWC3, which is located at chromosome xq22, was identified. The expression level of circWWC3 was significantly elevated in TNBC specimens compared with that in corresponding adjacent tissues based on tissue microarray analysis. This finding was corroborated by the RNA-sequencing results. Wang et al. have demonstrated that circWWC3 expression is significantly upregulated in clear cell renal cell carcinoma tumor tissues compared with that in adjacent normal tissues, whilst showing significant association with both T stage and pathological grade (29). Furthermore, the association between circWWC3 expression and various clinicopathologic features was analyzed. Elevated circWWC3 levels in TNBC tissues were found to be markedly associated with larger tumor size and lymph node metastasis. These clinical features indicate a close relationship with tumor progression. According to the aforementioned clinical significance, the biological functions of circWWC3 in TNBC were further investigated using both gain-of-function and loss-of-function strategies. circWWC3 was observed to enhance the proliferation, migration and invasion capabilities of TNBC cells.

Previous studies have shown that circRNAs can participate in the malignant progression of tumors by activating various signaling pathways, such as the PI3K/AKT/mTOR signaling pathway (30), Janus kinase/STAT pathway (31), NF-κB pathway (32), Ras signaling pathway (33), and TGF-β signaling pathway (34). To explore the downstream signaling pathways associated with circWWC3, mRNA expression profile microarray analysis we conducted, revealing that circWWC3 serves a role in the regulation of various processes, including cytokine-cytokine receptor interactions, tight junctions and natural killer cell-mediated cytotoxicity. Among potential downstream targets, we focused on CSF2 (granulocyte-macrophage colony-stimulating factor, GM-CSF), a cytokine that promotes stem cell proliferation and differentiation (35). Numerous studies have suggested that CSF2 can serve a significant role in promoting tumor growth and progression. This cytokine is involved in regulating cancer cell proliferation and migration across different cancer types, where its continuous expression and secretion are associated with tumor development and poor prognosis in various cancer models (22, 36, 37). In the present study, the overexpression of circWWC3 was revealed to influence the expression of the downstream target gene CSF2, which supports the development of TNBC. Therefore, circWWC3 likely serves a role in the malignant progression of TNBC, at least in part through the secretion of CSF2.

The functions of specific circRNAs are largely determined by their subcellular localization. crcRNAs located in the nucleus may function as cis-acting transcription regulators (38). whilst those found in the cytoplasm can serve as miRNA sponges (39, 40) or bind to RNA-binding proteins (41, 42). In the present study, circWWC3 was found to be predominantly found in the cytoplasm. The interaction between vimentin and circWWC3 was identified through RNA pull-down and mass spectrometry, which was subsequently verified by Western blotting. Vimentin, a type III intermediate filament protein, is a well-established marker of mesenchymal cells during EMT. It serves a crucial role in preserving cell morphology and enhancing cytoskeletal interactions. Moreover, vimentin functions as a scaffold for various important proteins that are involved in cell adhesion and migration (43). It has been shown to interact with several proteins, including chemokine-like factor super family 6, Mitogen-Activated Protein Kinase Kinase 4 and Sequestosome 1 (p62), to facilitate invasion in various types of malignant tumors (44–46). Furthermore, emerging evidence indicates that vimentin exhibits immunomodulatory functions beyond its structural roles. A study has found that vimentin can directly serve as an endogenous ligand for the Dectin-1 receptor, triggering the Syk signaling pathway and promoting the secretion of IL-1β (47). Additionally, it has been reported that tumor cells may evade NK cell immune surveillance through vimentin-mediated cytoskeleton remodeling (48). Our study further revealed that knockdown of circWWC3 enhanced the cytotoxic activity of NK cells, whereas this promotive effect was abolished upon vimentin overexpression. These findings suggest that circWWC3 modulates NK cell-mediated cytotoxicity through a mechanism involving vimentin function.

A previous study has indicated that circRNA can interact with vimentin to influence cellular biological functions. Circ-protein tyrosine kinase 2 (circPTK2) was found to enhance vimentin expression by physically binding to its phosphorylation sites at Ser38, Ser55, and Ser82, and was involved in the metastasis of colorectal cancer (23). Similarly, circKEAP1-encoded KEAP1-259aa binds cytoplasmic vimentin and promotes its ARIH1-mediated proteasomal degradation, suppressing osteosarcoma progression. circKEAP1 also activates RIG-I–dependent IFN-γ signaling to stimulate antitumor immunity (49). Notably, Golgi-protein 73 (GP73) interacts with vimentin and promotes PP1A-mediated dephosphorylation at Ser56, facilitating vimentin polymerization and inhibiting TRIM56-dependent ubiquitination and degradation (50).

In line with these findings, we discovered that circWWC3 binds vimentin at the S56 phosphorylation site, potentially regulating vimentin function through a comparable mechanism.

CSF2 is increasingly recognized as an important immunomodulator in cancer therapy. A previous study showed that a combination of PD-1 blockade, radiotherapy, and GM-CSF can induce profound and durable regression in chemo-refractory MSS/pMMR metastatic colorectal cancer (51). Furthermore, studies have indicated that GM-CSF-driven myeloid-derived suppressor cells (MDSCs) may upregulate prostaglandin E2 (PGE2) expression, thereby suppressing the effector functions of macrophages, cytotoxic T cells, and NK cells, while simultaneously promoting pro-tumorigenic Th2, Th17, and regulatory T cell (Treg) responses (52), which further facilitate tumor immune evasion and progression. In our study, circWWC3 was found to promote the progression of triple-negative breast cancer (TNBC). The underlying mechanism may involve enhanced CSF2 secretion following circWWC3 overexpression, thereby facilitating the formation of an immunosuppressive tumor microenvironment.

Previous studies have confirmed that the key EMT protein vimentin can influence the expression of various downstream genes (53–55). Additionally, the EMT in breast cancer cells can enhance the secretion of CSF2 (24). The present study also found that knockdown of circWWC3 can inhibit EMT and the secretion of CSF2, where overexpression of vimentin can reverse this phenomenon. Subsequently, the role of circWWC3 in the progression of TNBC *in vivo* was next investigated by creating a TNBC xenograft model. The knockdown of circWWC3 was found to significantly inhibit the growth of xenograft tumors. However, this inhibitory effect was reversed upon the overexpression of vimentin. As a result, these results suggest that circWWC3 can interact with vimentin to promote the EMT signaling pathway, leading to the increased secretion of CSF2 and accelerating the malignant progression of TNBC.

However, there were several limitations to the present study. First, our clinical dataset lacked survival data, which prevented an analysis of the relationship between circWWC3 and the survival of triple-negative breast cancer patients. Second, although our study focuses on the interaction between circWWC3 and vimentin, we acknowledge that the upregulation of CSF2 may involve other mechanisms. For instance, transcriptional regulation of CSF2 has also been linked to signaling pathways such as JAK/STAT (56) and NF-κB (57).

In conclusion, findings from the present study indicate that elevated expression levels of circWWC3 serve a role in the malignant progression of TNBC by directly interacting with the phosphorylation site S56 of vimentin, and this interaction is associated with increased secretion of CSF2. These findings suggest that circWWC3 may serve as a potential biomarker for TNBC, though further mechanistic studies and clinical validation are required to establish its translational applicability.

## Data Availability

The data presented in the study are deposited in the NCBI Sequence Read Archive repository, accession number PRJNA1310181; in the Gene Expression Omnibus repository, accession number GSE306483; and in the PRIDE Archive repository, accession number PXD067788.

## Supplementary material

Supplementary Figure 1 Transfection efficiency of circWWC3 overexpression and knockdown. **(A)** The knockdown efficiency of circWWC3 was demonstrated in MDA-MB-231 and BT-549 cells transfected with small interfering RNA circWWC3. **(B)** The overexpression efficiency of circWWC3 was exhibited in MDA-MB-468 and HS-578T cells transfected with the circWWC3 overexpression vector. **P<0.01. Circ, circular RNA.

Supplementary Figure 2 The impact of WWC3 on breast cancer patient prognosis was evaluated using the Kaplan-Meier plotter database.
