# Supplementary figures and images for: circWWC3 enhances the progression of triple-negative breast cancer by interacting with vimentin to regulate the secretion of CSF2
Source: Front Immunol. 2025 Oct 8;16:1665608. doi: 10.3389/fimmu.2025.1665608 (PMC12540408; doi:10.3389/fimmu.2025.1665608)

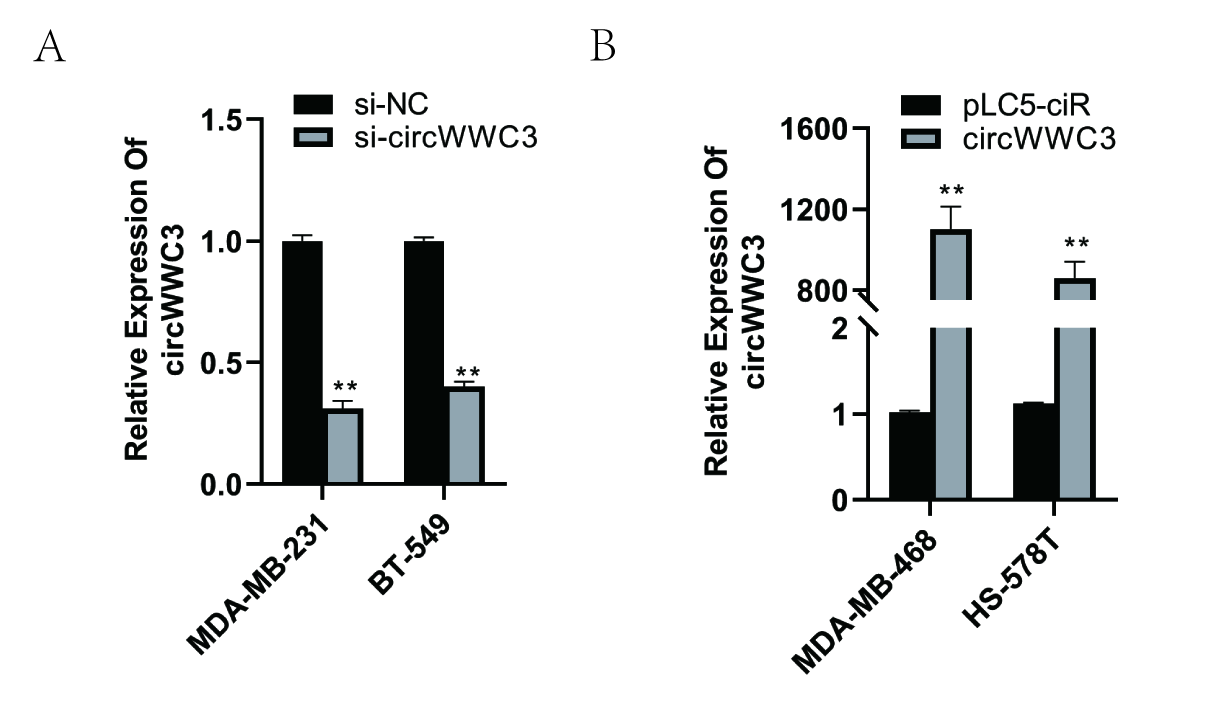

Supplement: Supplementary Figure 1 — Transfection efficiency of circWWC3 overexpression and knockdown. (A) The knockdown efficiency of circWWC3 was demonstrated in MDA-MB-231 and BT-549 cells transfected with small interfering RNA circWWC3. (B) The overexpression efficiency of circWWC3 was exhibited in MDA-MB-468 and HS-578T cells transfected with the circWWC3 overexpression vector. **P<0.01. Circ, circular RNA. [file Image1.tif]

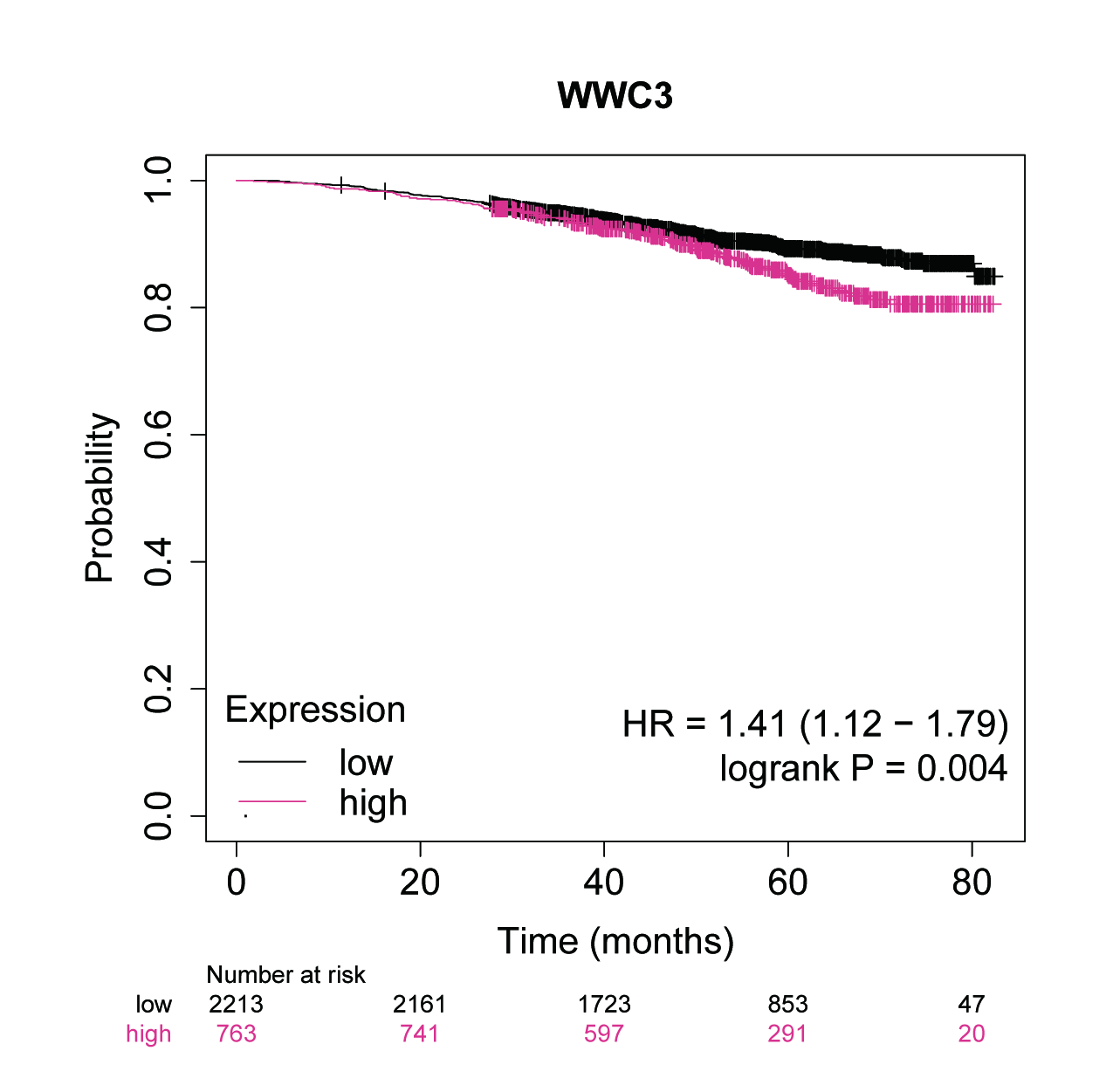

Supplement: Supplementary Figure 2 — The impact of WWC3 on breast cancer patient prognosis was evaluated using the Kaplan-Meier plotter database. [file Image2.tif]
